# Cross-sectional study of soluble selectins, fractions of circulating microparticles and their relationship to lung and skin involvement in systemic sclerosis

**DOI:** 10.1186/s12891-015-0653-8

**Published:** 2015-08-12

**Authors:** Line V. Iversen, Susanne Ullman, Ole Østergaard, Christoffer T. Nielsen, Poul Halberg, Tonny Karlsmark, Niels H.H. Heegaard, Søren Jacobsen

**Affiliations:** Department of Dermatology, Copenhagen University Hospital, Bispebjerg Hospital, Copenhagen, 2200 Denmark; Department of Autoimmunology & Biomarkers, Statens Serum Institut, Copenhagen, 2300 Denmark; Department of Clinical Biochemistry & Pharmacology, Odense University Hospital, Odense, 5000 Denmark; Department of Rheumatology, Rigshospitalet, Copenhagen University Hospital, Blegdamsvej 9, Copenhagen, 2100 Denmark

## Abstract

**Background:**

Endothelial damage and activation may play central roles in the pathogenesis of systemic sclerosis (SSc) and are reflected by microparticles (MPs) and soluble selectins. The objective of this study was to determine if these potential biomarkers are associated with specific organ involvements or cutaneous subgroups of SSc patients.

**Method:**

MPs in platelet-poor plasma from 121 patients with SSc, 79 and 42 with limited and diffuse cutaneous disease, respectively, were characterized by flow cytometry for their capacity to bind annexin V in combination with surface markers of either platelets (PMPs), leukocytes (LMPs) or endothelial cells (EMPs). Soluble E- and P-selectin levels were determined in plasma. By correlation analyses, this was held against involvement of skin, lung function, lung fibrosis, pulmonary artery hypertension, and serology.

**Results:**

None of the markers were associated with cutaneous subgroups of SSc. Concentrations of annexin V non-binding EMPs and annexin V non-binding LMPs were negatively correlated to pulmonary diffusing capacity (DL_CO_) (r = -0.28; p = 0.003; r = -0.26; p = 0.005) and forced vital capacity (FVC) (r = -0.24; p = 0.009; r = -0.29; p = 0.002), driven by patients with limited and diffuse cutaneous disease, respectively. Soluble E-selectin levels correlated negatively to DL_CO_ (r = -0.21, p = 0.03) and FVC (r = -0.25; p = 0.007); and soluble P-selectin correlated negatively to DL_CO_ (r = -0.23, p = 0.01).

**Conclusion:**

Negative correlations between annexin V non-binding EMP and LMP concentrations with lung function parameters (DL_CO_ and FVC) differed between limited and diffuse cutaneous subsets of SSc, indicative of various pathogeneses of lung involvement in SSc, possibly with a differential role of MPs.

## Background

Systemic sclerosis (SSc; scleroderma) is a systemic autoimmune connective tissue disease of unknown etiology, characterized by vasculopathy and fibrotic changes in the skin and various internal organs. Clinically, SSc is classified according to the extent of skin involvement in a limited cutaneous (lcSSc) and a diffuse cutaneous (dcSSc) type, which may have different pathogenesis, clinical courses and prognoses [1].

A major part of the increased mortality of patients with SSc is due to lung involvement, i.e. interstitial lung fibrosis and/or pulmonary arterial hypertension (PAH) [2]. Isolated PAH is more common in lcSSc [3, 4] and lung fibrosis with or without PAH is more frequent in dsSSc [5, 6]. Thus, lung function tests showing an isolated reduction of the diffusing capacity is most commonly seen in lcSSc while restrictive lung disease is more typical of dcSSc [3, 4, 7]. These two patterns of abnormal lung function may represent different types of pathogenesis rather than different stages of development.

Vasculopathy with perivascular inflammation and signs of coagulation activation is an early and central event in the pathogenesis of SSc [8]. Accordingly, several soluble markers of endothelial damage are increased in the circulation of SSc patients such as soluble selectins, soluble intercellular adhesion molecule 1, soluble vascular cell adhesion molecule 1, thrombomodulin, von Willebrand factor protein and endothelin-1 [8, 9]. Other markers of endothelial damage may be subcellular particles, microparticles (MPs) [10] which are most often arbitrarily defined according to size, 0.1-1.0 μm in diameter, but may be even larger. Generation and shedding of MPs occur during biological processes of considerable diversity, including normal cellular differentiation, or apoptotic cell breakdown, shear stress as present in arteries with severe stenosis and during cellular activation following stimulation with proinflammatory or prothrombotic substances [11]. The membrane and membrane proteins of MPs reflect their cellular origin allowing differentiation into *e.g.* MPs derived from platelets (PMP), leukocytes (LMP), or endothelial cells (EMP). The composition and content of MPs likely reflect both the type and state of their parent cells or tissues and make them potentially valuable markers of inflammation or vascular reactivity.

The fraction of MPs, which does not bind annexin V, AnxV non-binding MPs (AnxV^−^ MPs), has been shown increased in SSc patients, whereas concentrations of AnxV positive MPs (AnxV^+^ MPs) did not differ from healthy controls [12]. We therefore only correlated AnxV^−^ MP counts to disease manifestations and indicators of disease activity in SSc and found AnxV^−^ MPs subsets to be associated with impaired lung function. The AnxV^−^ MPs most likely originate from activated cells since their concentrations correlated with markers of cellular activation (soluble E-selectin (sE-selectin, sCD62E) and soluble P-selectin (sP-selectin, sCD62P)) in SSc [12]. Correlation of sE-selectin and sP-selectin with lung function parameters in SSc patients may reflect activation of the lung endothelium in SSc lung disease. The sE- and sP-selectins are released from activated platelets and endothelial cells, and as for the AnxV^−^ MPs, the contribution of selectins released in excess from the lungs may contribute to the observed correlation with lung function.

## Methods

This cross-sectional study included 121 consecutive patients fulfilling American College of Rheumatology criteria [13] for SSc; 102 women and 19 men. Lung fibrosis was not an inclusion criterion. Other aspects of this patient population have been reported previously [12]. All but one patient were Caucasians. Eight patients received azathioprine, methotrexate, penicillamin, or cyclosporine and 6 were given prednisolone at the time of inclusion. None had received therapy with biological drugs or prostanoid infusion within 6 month of inclusion. Concomitant treatment of SSc patients included among others calcium channel blockers, proton pump inhibitors, angiotensin-converting enzyme inhibitors, diuretics, statins and non-steroidal anti-inflammatory drugs. Patients with cancer, pregnancy or systemic infections were excluded. The study was approved by the ethics committee of the capital region of Copenhagen (approval number H-B-2008-131) and carried out in accordance with the principles of the Declaration of Helsinki. All participants were included after giving written informed consent.

### Clinical, biochemical and serological assessment

The clinical characteristics of the patients are summarized in Table 1. Data on disease history including clinical, serological and pharmacological data were obtained by medical records, patient examination and interview. Sub classification into limited and diffuse cutaneous disease was done according to LeRoy et al. [14].

**Table 1:** Demographical and clinical characteristics of 121 patients with systemic sclerosis

Patients	All	lcSSc	dcSSc
Gender, males/females, n	19/102	3/76	16/26
Age, mean ± SD (range) years	57 ± 12 (22-79)	59 ± 11	55 ± 12
Disease duration, mean ± SD (range) years	12 ± 9 (0-53)	12 ± 9	11 ± 9
Cutaneous Involvement			
ᅟModified Rodnan skin score, mean ± SD	11 ± 9	7 ± 3	20 ± 9
ᅟCutaneous ulcers, all, n	68	44	24
ᅟCutaneous ulcers, present, n	23	10	13
ᅟCutaneous ulcers, past, n	45	34	11
ᅟCalcinosis, n (N)*	43 (107)	30	13
Pulmonary and Cardiac involvement			
ᅟDL_CO_, mean ± SD % predicted (N)*	64.6 ± 19.9 (118)	64.2 ± 18.5 (77)	66.3 ± 20.3 (41)
ᅟPatients with DL_CO_ < 80 (%)	75 %	79 %	74 %
ᅟFVC, mean ± SD % predicted (N)*	95.7 ± 21.1 (118)	99.3 ± 20.1 (77)	89.1 ± 21.7 (41)**
ᅟPatients with FVC < 80 (%)	22 %	20 %	27 %
ᅟRadiographically confirmed lung fibrosis, n	21	14	7
ᅟElevated SPAP by echocardiography, n (N)*	10 (115)	6	4
Serology			
ᅟAnti-nuclear antibodies, n	116	74	42
ᅟAnti-Scl-70 antibodies, n	15	3	12
ᅟAnti-centromere antibodies, n	49	41	8
ᅟPeripheral blood			
ᅟLeukocytes total, mean ± SD (x 10^9^/L)	7.2 ± 2.2	7.4 ± 2.5	7.1 ± 2.3
ᅟNeutrophils, mean ± SD, (x 10^9^/L)	4.6 ± 1.8	4.7 ± 2.0	4.4 ± 1.6
ᅟPlatelets, mean ± SD, (x 10^9^/L)	275 ± 86	265 ± 86	268 ± 72
Others			
ᅟDMARDS, n	8	4	4
ᅟCurrent or previous smokers, n	83	57	26
ᅟArterial hypertension, n	30	18	12

dcSSc: diffuse cutaneous systemic sclerosis; lcSSc: limited cutaneous systemic sclerosis; anti-Scl-70: anti-topoisomerase-1; DMARDS: disease modifying anti rheumatic drugs; arterial hypertension: blood pressure above 140/90 mmHg at study inclusion; DL_CO_: carbon monoxide diffusing capacity in percent of predicted values; FVC: vital capacity in percent of predicted values. n: number of subjects with feature. N: total number of subjects in the group. *: reduced number of patients due to lack of information. **, *p* < 0.01

Disease duration was defined as time since first non-Raynaud SSc manifestation. All but one patient reported the occurrence of Raynaud’s phenomenon after exposure to low temperatures. At inclusion, the modified Rodnan skin score was assessed in 17 body areas by the same experienced clinician [15]. Cutaneous ulcers were defined as fingertip ulcers or other ulcers and registered as present at enrollment or past ulcers. Subcutaneous calcinosis was demonstrated by X-ray of hands and/or feet present at enrollment.

The lung function was determined within one year of patient-inclusion by means of standard lung function tests, including forced vital capacity (FVC) and diffusing capacity of carbon monoxide (DL_CO_) measured by the single-breath CO and helium CO-dilution technique standardized for hemoglobin level. FVC and DL_CO_ were reported in percent relative to predicted values with respect to sex, age, height, and weight [16] and DLco- and FVC-values lower than 80 % were considered pathological [17]. Isolated reduction of DL_CO_ was defined as DL_CO_ < 80 % of the predicted value with FVC > 80 % of the predicted value. Lung fibrosis was defined as radiographic signs of lung fibrosis demonstrated by chest X-ray, which in 7 cases was supplemented with high resolution computed tomography (HRCT).

Elevated systolic pulmonary arterial pressure measured by echocardiography was defined as an estimated pressure above 40 mmHg [18] and arterial hypertension was defined as a blood pressure above 140/90 mmHg at study inclusion.

Antinuclear antibodies (ANA) were detected by indirect immunofluorescence technique on HEp-2 cells. Scl-70-antibodies were demonstrated by ELISA. Routine laboratory parameters were determined at the local department of clinical biochemistry.

### Blood sampling and isolation of platelet-poor plasma

Venipuncture was performed with a 21-gauge needle, and after release of the tourniquet the first tube was obtained and always reserved for serological analyses. Next, for microparticle isolation blood was collected into 3 x 9 mL citrate tubes (Vacuette sodium citrate 3.8 %, Greiner Bio-One, Kremsmünster, Austria) which were gently mixed 5 times. Immediately after collection, blood cells were removed by centrifugation. The samples underwent a 2 step centrifugation procedure: 1800 g, 10 min, 21 °C, followed by a second centrifugation of the supernatant: 3000 g, 10 min, 21 °C, to obtain platelet poor plasma (PPP). The PPP was aliquoted, snap-frozen in liquid nitrogen, and stored at -80 °C until analysis [19, 20].

### Analysis of MPs by flow cytometry

MPs, here defined as particles smaller than 1 μm, were measured directly in platelet poor plasma [20]. In brief, a panel of cell-specific monoclonal murine antibodies was applied to label MPs originating from platelets (CD42a), leukocytes (CD45) and endothelial cells (CD146). The antibodies were used in the following formats and final concentrations after titration experiments: anti-CD42a-FITC (IgG_1_, 32 ng/mL, Becton-Dickinson, clone ALMA16), CD45-PE (IgG_1_, 65 ng/mL, Becton-Dickinson, clone HI30) and anti-CD146-FITC (IgG_1_, 262 ng/mL, AbD Serotec, clone OJ79c). Labeling of MPs by specific antibodies was compared to MPs labeled by isotype-matched control antibodies (IgG_1_-FITC, Becton-Dickinson, clone MOPC-21; IgG_1_-PE Becton-Dickinson, clone MOPC-21) at the same final concentration to set gate for positive events. AnxV-binding to MPs was measured using AnxV-APC (10 ng/mL final concentration, Becton-Dickinson) in the presence of 1 mM Ca^2+^. Platelet poor plasma aliquots (250 μL) were thawed on melting ice. Labeling of MPs for flow cytometric analysis was carried out by mixing 5 μL prediluted AnxV-APC and 5 μL prediluted specific antibodies or isotype-matched control antibodies with 5 μL heparin-sodium salt 10 % w/v (Sigma-Aldrich 194 USP/mg dry basis. Heparin was added to avoid clotting of the plasma). Finally, 5 μL PPP was added followed by dilution with 935 μL low phosphate buffered saline containing calcium, (PBS-Ca; 154 mmol/L NaCl, 1.4 mmol/L phosphate, pH 7.4, 2.5 mM CaCl_2_) and incubation for 1 hour in the dark. As a negative control experiment for AnxV binding, low phosphate buffered saline containing citrate (PBS-citrate; 154 mmol/L NaCl, 1.4 mmol/L phosphate, pH 7.4, 10.5 mM trisodium citrate) was used for dilution replacing the PBS-Ca.

To reduce background noise, buffers were filtered through 0.1 μm pore size filters (MiniSart HF, Sartorius Stedim Biotech S.A., Aubagne, France). Fresh buffers were prepared on a weekly basis or more frequently if a rise in background noise was observed indicating contamination. The samples were analyzed using a FACS Calibur flow cytometer (BD Biosciences) controlled by CellQuest software version 5.1.1 in the “high” flow rate mode. Flow rate was measured before each experiment. Both forward scatter (FSC) and side scatter (SSC) were recorded with logarithmic gain. Acquisition time was 60 seconds. MP gating was accomplished using 1 μm beads (Flow Cytometry Size Calibration Kit, Molecular Probes, Inc., Eugene, OR, USA) for setting upper limits in both FSC and SSC signals, and a lower limit was placed to exclude buffer noise. The MP gate was validated by showing that the majority of AnxV^+^ MPs induced from platelets using the calcium ionophore A23187 (Sigma, Saint Louis, MO, USA) were detected using this gate (data not shown). MPs gated this way were further analyzed in SSC/FL-1, SSC/FL-2 or SSC/FL-4 plots to discriminate labeled particles from unlabeled particles using a fluorescence threshold determined by the fluorescence level of the MPs stained using the isotype-matched control antibodies and AnxV non-binding controls. Instrument settings were as previously described [12]. Flow cytometric data analysis was performed using FlowJo software version 7.6.1 (Tree Star, Inc., Ashland, OR, USA).

MP-plasma concentrations of MPs were calculated as MPs/mL based on MP count per unit time, flow rate of the flow cytometer and net dilution during sample preparation of the analyzed sample. All samples were double-labeled. The total cell derived population was calculated as the sum of all the cell derived MP populations (EMPs, LMPs and PMPs).

### Selectins

Plasma concentrations of soluble E-selectin (sE-selectin, sCD62E) and soluble P-selectin (sP-selectin, CD62P) were measured using enzyme-linked immunosorbent assay kits (Quantikine; R&D Systems; Minneapolis, MN). Serum samples were diluted 1:10 for sE-selectin and 1:20 for sP-selectin assays before analysis.

### Statistical analysis

Pearson’s correlation analysis was used to examine relationships between MP concentrations, lung function tests, and estimated pulmonary arterial pressure and clinical blood samples. Data were logarithmically transformed allowing parametric tests. In order to compare MP concentrations between patients with and without lung fibrosis Student’s t-tests were performed on logarithmically transformed data. Student’s t-test was used to compare the distribution of different clinical features in the different SSc patient subsets. Statistical significance was defined as p < 0.05. GraphPadPrism v. 5 and Microsoft Excel 2010 were used for the statistical calculations and plots. Data on MP concentrations and soluble selectin levels [12] are here correlated with clinical parameters,

## Results

We find no correlations between levels of MPs or soluble selectins and modified Rodnan skin score in the whole patient cohort or in subgroups. Also, no associations were found between MP concentrations and the following variables: autoantibodies, pulmonary arterial hypertension (PAH), digital ulcers neither present at enrolment nor only in the past, disease duration, smoking status, age, hypertension, IgM rheumatoid factor, C-reactive protein, or use of the following medications: low dose aspirin, calcium channel blockers and/or statins. SSc patients with calcinosis had a significantly lower concentration of AnxV^−^ EMPs (p = 0.04, Table 3).

### Lung function and circulating E- and P-selectins

**Fig. 1:**
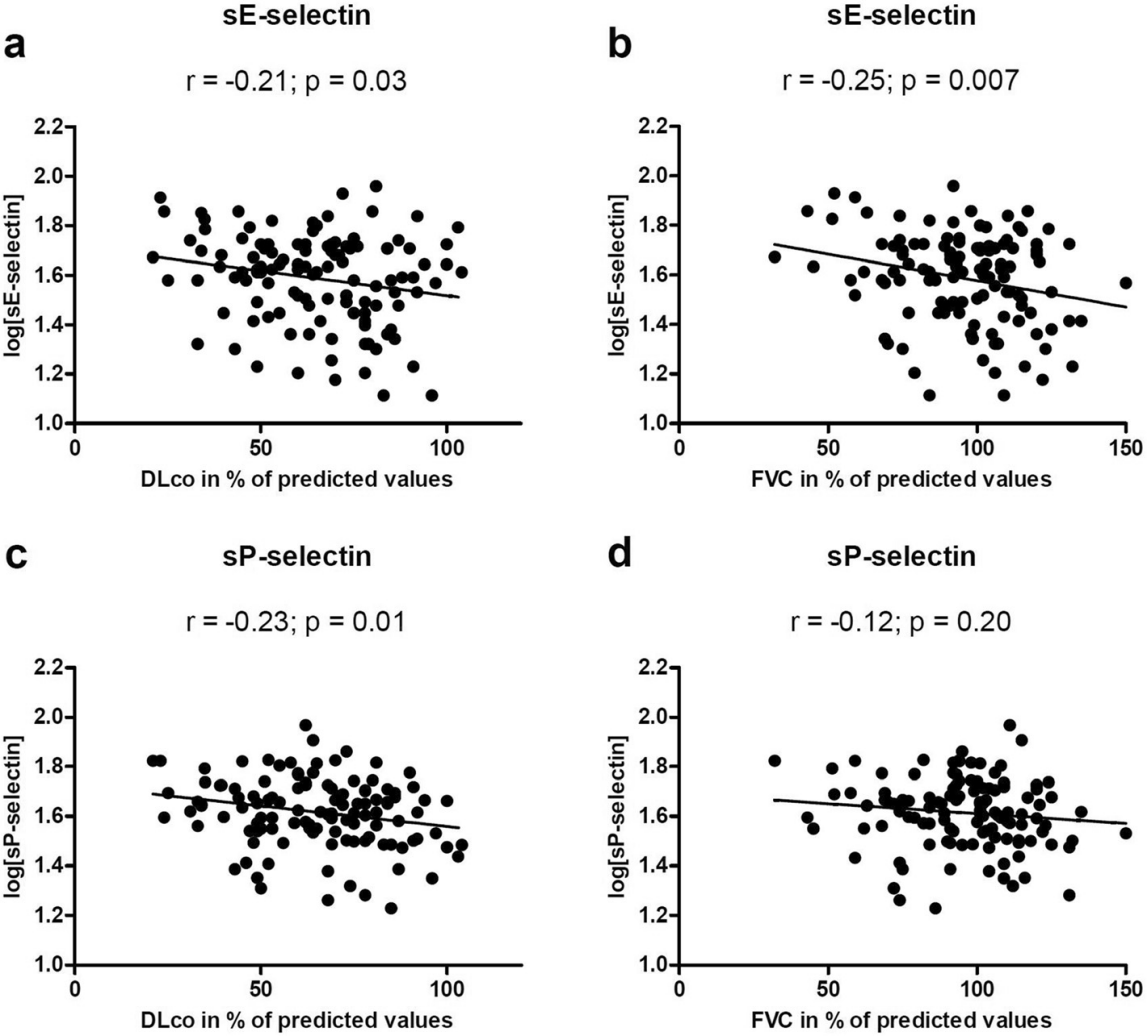
Plasma levels of soluble E- and P-selectins correlate with lung function parameters in systemic sclerosis patients. Soluble E-selectin (log ng/mL) correlated with (**a**) carbon monoxide diffusing capacity in percent of predicted values (DLCO %) (r = -0.21; p = 0.03) and (**b**) forced vital capacity in percent of predicted values (FVC %) (r = -0.25; p = 0.007). P-selectin (log ng/mL) correlated with (**c**) DLCO % (r = -0.23; p = 0.01) but not with (**d**) FVC %. Correlation analysis was performed by use of Pearson’s r test using log transformed selectin-levels

Soluble E- and P-selectins are markers for damaged and/or activated endothelium and platelets and have previously been shown to be elevated in SSc patients compared to healthy controls, nevertheless no objective measures of lung function were reported in these studies [12, 21–24]. Here data on lung function (vital and diffusing capacity) were available for 118 patients (Table 1). While diffusing capacity (DL_CO_) did not differ between the lcSSc (n = 77) and the dcSSc (n = 41) subgroups, there was a highly significant (*p* < 0.01) decreased vital capacity (FVC) in the dcSSc group (Table 1). Other studies have shown both equal and lower FVC and DL_CO_ in dcSSc compared with lcSSc patients [25, 26]. In our cohort, the DL_CO_ was reduced in both patient subgroups (average of all 118 patients was 65 % ± 20 % (1 SD)) and FVC was lower in the dcSSc group (99 % *vs.* 89 %) compared with the lcSSc group. Soluble E-selectin correlated inversely with measures of lung function, both with FVC and DL_CO_ (r = -0.25; p = 0.007 and r = -0.21; p = 0.03, respectively) (Fig. 1) and soluble P-selectin correlated with DL_CO_ (r = -0.23; p = 0.012). Thus, higher levels of these circulating selectins were found in patients with decreased lung function irrespectively of lcSSc or dcSSC status. As expected [27], soluble P-selectin correlated with platelet counts (r = 0.34, p = 0.001) but also with the neutrophil count (r = 0.27; p = 0.003). Also, soluble E-selectin was significantly increased (p = 0.006) in the 21 patients with chest x-ray confirmed lung fibrosis.

### Lung function and circulating microparticles

**Fig. 2:**
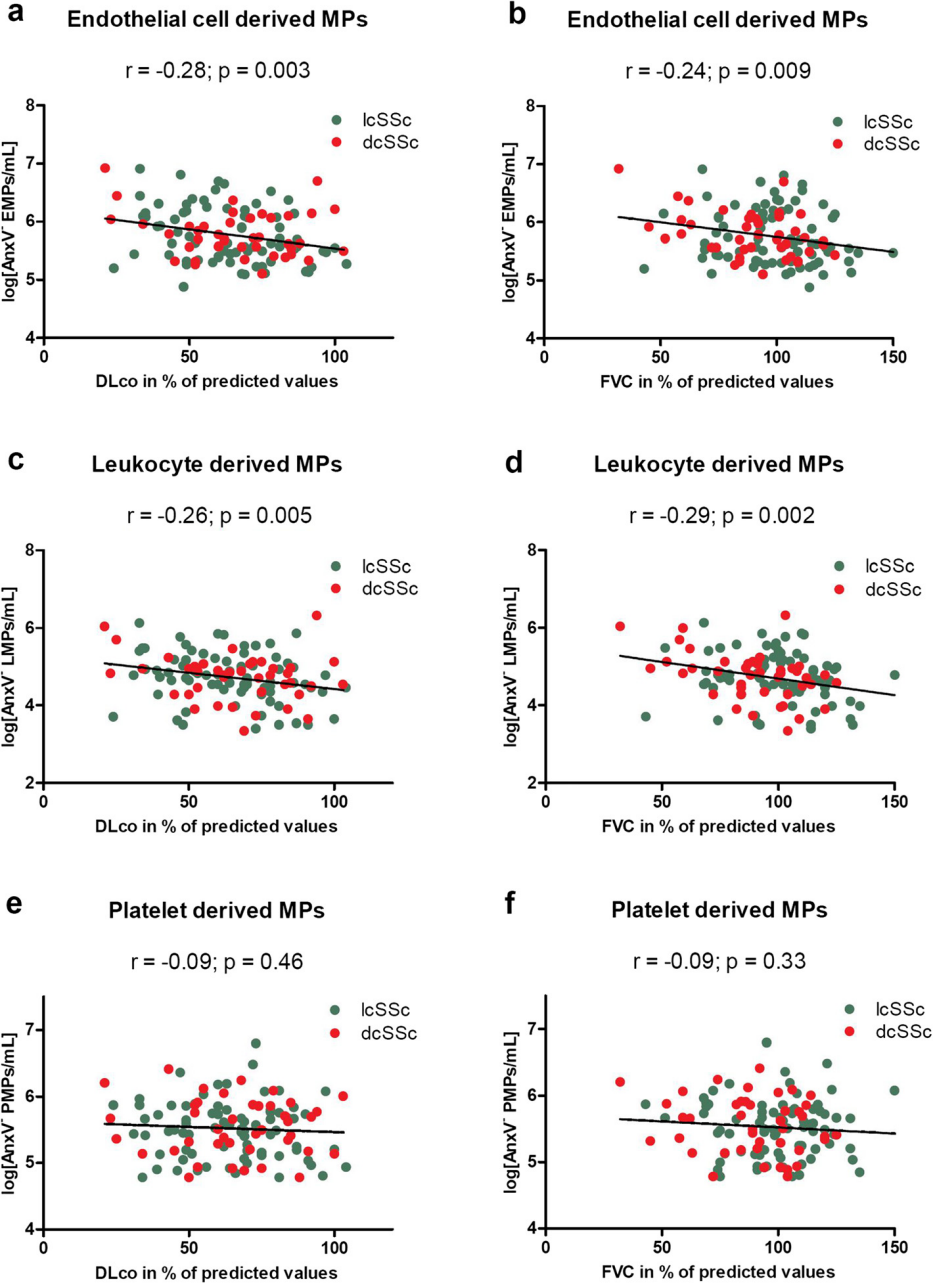
Plasma levels of annexin V non-binding, cell-derived microparticles (AnxV^−^ MPs) correlated to lung function parameters. Endothelial cell derived MPs and leukocyte derived MPs (log counts/mL) correlated with both carbon monoxide diffusing capacity in percent of predicted values (DLco, %) (r = -0.28; p = 0.003, and r = -0.26; p = 0.005) respectively (**a** and **b**) and forced vital capacity in percent of predicted values (FVC, %) (r = -0.24; p = 0.009, and r = -0.29; p = 0.002) respectively (**c** and **d**). No correlations between AnxV^−^ platelet derived MPs and lung function parameters were found (**e** and **f**). Correlation analysis was performed by use of Pearson’s r test using log transformed microparticle data

Circulating microparticles (MPs) were enumerated and phenotyped using flow cytometry on platelet-poor plasma samples from all 121 patients. MPs were probed for Annexin V binding, and for the expression of endothelial (CD146), leukocyte (CD45), and platelet (CD42a) markers. AnxV^−^ MPs were inversely correlated to lung function parameters for MPs derived from endothelial cells and leukocytes but not for platelet-derived MPs (Fig. 2) (DL_CO_, r = -0.28; p = 0.003, and r = -0.26; p = 0.005, respectively; FVC, r = -0.24; p = 0.009, and r = -0.29; p = 0.002, respectively). The correlation with FVC was driven by the dcSSc subgroup where the correlation was highly significant (p < 0.01). The correlation with DL_CO_ appeared to be driven by the lcSSc subgroup while the DL_CO_ and AnxV^−^ MPs were not significantly correlated in the dcSSc subgroup (Table 2). Both AnxV^−^ EMPs and AnxV^−^ LMPs correlated with neutrophil counts (r = 0.30; p = 0.001 and r = 0.24; p = 0.008, respectively). For the 21 cases with chest x-ray confirmed lung fibrosis we found an average of 94 % increased concentration of AnxV^−^ EMPs compared to cases without fibrosis (p = 0.02, Table 3). This association was driven by the 7 patients in the dcSSc group (p = 0.02 compared with dcSSc patients without lung fibrosis (n = 35)) as MP levels did not differ in the lcSSc patients with and without lung fibrosis.

**Table 2:** Correlation of annexin V non-binding (AnxV^−^) MP levels to lung function parameters in patients with systemic sclerosis

	Lung function			
	DLco		FVC	
	dcSSc	lcSSc	dcSSc	lcSSc
	(n =41)	(n = 77)	(n =41)	(n = 77)
	r	R	r	R
log(AnxV^−^ EMPs)	−0.21	−0.32**	−0.43**	−0.15
log(AnxV^−^ LMPs)	−0.22	−0.28*	−0.42**	−0.19
log(AnxV^−^ PMPs)	−0.06	−0.08	−0.18	−0.03

dcSSc: diffuse cutaneous systemic sclerosis; lcSSc: limited cutaneous systemic sclerosis. DL_CO_: Diffusing capacity for carbon monoxide. FVC: Forced vital capacity. Pearson’s r on log transformed data. P < 0.05*. P < 0.01**

**Table 3:** Concentrations of annexin V non-binding (AnxV^−^) MP subsets stratified by the presence of selected disease manifestation of systemic sclerosis (SSc)

Clinical Variable	AnxV^−^ EMPs					AnxV^−^ LMPs					AnxV^−^ PMPs				
	(MP x 10^5^)					(MP x 10^4^)					(MP x 10^5^)				
	Symptom		No symptom		P	Symptom		No symptom		p	Symptom		No symptom		p
	Mean	± SD	Mean	± SD		Mean	± SD	Mean	± SD		Mean	± SD	Mean	± SD	
**Skin**															
Cutaneous ulcer	8.01	±6.83	11.48	±16.11	0.64	9.47	±8.80	5.73	±31.49	0.84	4.59	±3.92	5.79	±7.88	0.66
Calcinosis	10.75	±9.71	9.16	±13.68	**0.04**	14.04	±18.78	14.59	±33.08	0.07	6.87	±10.39	4.41	±4.46	0.17
**Heart**															
SPAP > 40	18.46	±28.31	10.13	±13.10	0.63	24.64	±37.96	14.29	±27.95	0.96	4.63	±4.84	5.64	±7.54	0.43
**Lung**															
Fibrosis (among all)	18.06	±22.91	9.30	±12.26	**0.02**	23.67	±35.67	13.35	±27.08	0.14	5.29	±4.82	5.62	±7.79	0.83
Fibrosis (among dcSSc)	22.13	±28.29	9.43	±12.36	**0.02**	28.14	±39.47	13.46	±27.34	0.13	7.19	±6.97	5.49	±7.80	0.54
Fibrosis (among lcSSc)	16.03	±20.61	9.43	±12.36	0.12	21.44	±34.96	13.46	±27.34	0.40	4.34	±3.23	5.49	±7.80	0.87

Student's *t* test**:** significance level: p < 0.05 in bold

mRSS: modified Rodnan skin score. SPAP: Systolic pulmonary arterial pressure. dcSSc; diffuse cutaneous SSc. lcSSc: limited cutaneous SSc

## Discussion

In this study, we found the concentration of AnxV^−^ EMPs and AnxV^−^ LMPs to be inversely correlated with the lung function in SSc patients as measured by DL_CO_ and FVC. These correlations were dissimilar in patients with limited and diffuse disease. Even though the extent of cutaneous and pulmonary involvement in SSc are not directly correlated [5, 6] this difference may reflect an increased prevalence of restrictive lung disease in patients with dcSSc and increased prevalence of isolated pulmonary hypertension more often is seen in lcSSc [3]. The dissimilar correlation between MP counts in patients with dcSSc and lcSSc to FVC and Dlco, respectively, may also support the notion that different types of lung involvement exists in these two subsets of patients possibly related to different activation of coagulation and fibrinolysis. For instance, dermatan sulfate, which coats endothelial cells and modulates their adhesiveness and immunogenicity was only found increased in plasma from dcSSc and plasminogen activation inhibitor (PAI) was only elevated in lcSSc [21]. The associations between lung function and levels of AnxV^−^ EMPs and AnxV^−^ LMPs may reflect an increased activation of endothelial cells and leukocytes in the lungs. It has been shown that pulmonary microvascular endothelial cells and aortic endothelial cells *in vitro* generate diverse numbers and subtypes of EMPs in response to different proinflammatory stimuli [28]. Accordingly, the use of other endothelial cell markers than CD146 would be of interest to explore in future studies. Furthermore, HRCT, today mandatory in the diagnosis of ILD in SSc patients, would be beneficial to elaborate the connection between MPs, lung function test and lung fibrosis, as this examination is a more sensitive detector of the extent and severity of interstitial lung involvement [29].

Only few previous studies report on circulating AnxV^−^ MPs [12, 19, 30] and only one has characterized these MPs in SSc patients and found that the fraction of AnxV^−^ MPs was higher than in healthy controls [12]. Three groups have reported that circulating overall MP concentrations in SSc patients are equal or elevated compared with healthy controls [23, 31, 32]. One study found elevated numbers of PMPs in SSc-interstitial pneumonitis, but had no information about lung function or radiographic findings [23]. Another study found elevated PMP concentrations in SSc patients with PAH defined as values above 25 mmHg estimated by echocardiography. The PMP levels in these patients were also found to correlate with the increased pulmonary arterial pressures [31]. We do not reproduce these findings. We find no overall increased MP-concentrations and no MP-correlations to pulmonary arterial pressure (using an echo-pressure threshold of 40 mmHg). Right heart catheterization is however a more accurate method to asses PAH than transthoracic echocardiography, but the risk and discomfort associated with this test made it unacceptable to use in this study [33].

Other clinical connections reported are inverse correlation of total MPs and PMPs to modified Rodnan skin score, and lower total numbers of MPs and PMPs in patients with digital ulcers [31]. We could not confirm this. Only one other study reports a link between reduced lung function and EMPs which was found in healthy smokers compared to healthy controls. In this study, a reduced DL_CO_ was found to be inversely correlated with the concentration of EMPs in smokers [34].The reasons for these discrepancies are unknown but may be related to technical, physiological, and sample size parameters. Also, the size and the composition of the circulating MP population depend on several physiological variables including exercise, menstrual cycle, age, body mass index, and on countless pathologic conditions as well as on smoking and the intake of medicine. Consequently, it is necessary to study large numbers of patients to minimize the impact of biological/physiological variation that may interfere with the study results [35]. As to the selection of patients for this study, the mean disease duration of the patients was 12 years and only 3 patients did not have proximal cutaneous sclerosis, which indicated that the patients in this study were well into the disease process. Using the new 2013 ACR/EULAR criteria for SSc might have resulted in the inclusion of more patients with discreet symptoms or early SSc, which could have had implications for the results obtained. However, this study was performed before prior to the launching of these criteria [36, 37].

The concentration of AnxV^−^ MPs in fresh (never frozen), samples is likely to be higher than when using frozen plasma samples as freezing has been shown to induce increase phosphatidylserine exposure and thereby to increase binding of annexin V to all MPs [20, 38]. MPs that do not expose PS after freezing may well have specific characteristics which remain to be elucidated, as should more detailed studies of differences between fresh and frozen samples.

## Conclusions

In this study of a large number of clinically well-characterized SSc patients that were sampled under controlled and identical conditions, we find that levels of circulating AnxV^−^ EMPs and AnxV^−^ LMPs are inversely correlated with the lung function as measured by DL_CO_ and FVC. Worse lung function correlates with higher levels of Anx V non-binding MPs. These correlations are different in patients with limited and diffuse disease. In dcSSc the increased concentration of AnxV^−^ MPs from endothelial cells and leucocytes is related to a reduction of FVC, whereas in lcSSc the same MP compartment is associated with a reduction of DL_CO_. The observation that the concentration of AnxV^−^ EMPs was higher in patients with chest x-ray confirmed lung fibrosis merits further studies supported by HRCT and including longitudinal studies with consecutive sampling. Also, further characterization of the composition and origin of the unique MP populations, especially the MPs that do not bind annexin V, that circulate in SSc patients is required to increase our understanding of the putative role of MPs in the pathogenesis and disease activity monitoring in SSc.
